# Comprehensive evaluation of a multicenter real-world study: neoadjuvant immunochemotherapy in locally advanced gastric cancer

**DOI:** 10.1097/JS9.0000000000001645

**Published:** 2024-05-22

**Authors:** Kexun Li, Simiao Lu, Xuefeng Leng, Lin Peng

**Affiliations:** Department of Thoracic Surgery, Sichuan Clinical Research Center for Cancer, Sichuan Cancer Hospital and Institute, Sichuan Cancer Center, Affiliated Cancer Hospital of University of Electronic Science and Technology of China (Sichuan Cancer Hospital), Chengdu, People’s Republic of China

*Dear Editor,*

We have carefully read the paper titled ‘The safety and efficacy of neoadjuvant immunochemotherapy following laparoscopic gastrectomy for gastric cancer: a multicenter real-world clinical study’ by Sun *et al*.^1^ investigates the safety and efficacy of neoadjuvant immunochemotherapy (nICT) compared to neoadjuvant chemotherapy (nCT) in patients with locally advanced gastric cancer (LAGC). Through propensity score matching, 585 patients were analyzed, with 195 in the nICT group and 390 in the nCT group. Results indicate that the nICT group exhibited higher objective response rates (79.5% vs. 59.0%), pathological complete response rates (14.36% vs. 6.41%), and major pathological response rates (39.49% vs. 26.15%) compared to the nCT group. Surgical complications and perioperative outcomes did not significantly differ between the groups. Additionally, the nICT group had a lower rate of early recurrence compared to the nCT group (29.7% vs. 40.8%). Multivariable analysis identified immunotherapy as an independent protective factor against early recurrence. No significant variations were observed in neoadjuvant therapy drug toxicity. This real-world study suggests that nICT is a safe and effective approach, leading to improved radiological and pathological responses and reduced early recurrence rates in patients with LAGC. In contrast, Sun and colleagues’ study provides a valuable retrospective study comparing the outcomes of nICT versus nCT alone in patients with locally advanced or metastatic esophageal cancer. The authors’ analysis provides important insights, but several aspects warrant further consideration.

Firstly, Table 2 in the original article shows a statistically significant difference in the number of lymph nodes dissected between the nICT and nCT groups after propensity score matching. This variable is crucial, as the extent of lymph node dissection can significantly impact patient survival in tumors of the digestive tract. By ensuring balance in this key factor through matching, the authors have strengthened the validity of their findings^2,3^.

Secondly, the authors present the Kaplan–Meier curves for the cumulative incidence of any recurrence, comparing the matched nICT and nCT groups. While this analysis is informative, the authors should also consider examining the pre-matching Kaplan–Meier curves. If the two groups exhibited similar trends before matching, it would further bolster the conclusion that the addition of immunotherapy to nCT confers a survival advantage.

Lastly, the authors report on the recurrence patterns of the patients but do not provide details on the salvage treatments administered after recurrence. Post-recurrence management can significantly influence overall patient outcomes, particularly in this high-risk, advanced-stage population. It would be valuable for the authors to investigate the impact of subsequent therapies on survival, as this information could guide clinicians in optimizing treatment strategies for these patients^4,5^.

In summary, this multicenter real-world study offers valuable insights into the safety and efficacy of nICT versus nCT for patients with LAGC. Following propensity score matching, the study revealed that the nICT group exhibited significantly higher objective response rates, pathological complete response rates, and major pathological response rates compared to the nCT group. Moreover, the nICT group had a reduced risk of early recurrence, indicating better long-term outcomes. Overall, this well-designed study suggests that nICT is a safe and effective treatment option for LAGC patients, leading to improved radiological and pathological responses and a lower risk of early recurrence when compared to nCT. These results support further exploration and potential adoption of nICT as a promising neoadjuvant strategy for managing LAGC. The authors have conducted a well-designed study comparing nICT and nCT in esophageal cancer. Addressing the considerations raised in this commentary, such as the role of lymph node dissection, pre-matching survival trends, and post-recurrence management, could enhance the conclusions and provide more comprehensive insights for clinicians and researchers in this field.

## Ethical approval

Not applicable.

## Consent

Not applicable.

## Source of funding

This work was supported by grants from the National Key Research and Development Program (2022YFC2403400), the Department of Science and Technology of Sichuan Province (2023YFS0044, 2024YFHZ0322), and Sichuan Province Clinical Key Specialty Construction Project.

## Author contribution

All authors: study concept and design, acquisition, analysis, or interpretation of data; K.L.: drafting of the article and statistical analysis; L.P. and X.L.: administrative, technical, or material support and obtained funding; K.L., X.L., and L.P.: had full access to all the data in the study and take responsibility for the integrity of the data and the accuracy of the data analysis.

## Conflicts of interest disclosure

The authors declare no conflicts of interest.

## Research registration unique identifying number (UIN)

Not applicable.

## Guarantor

Kexun Li and Lin Peng are guarantors.

## Data availability statement

This study does not involve new data, only data from ‘The safety and efficacy of neoadjuvant immunochemotherapy following laparoscopic gastrectomy for gastric cancer: a multicenter real-world clinical study.’

## Provenance and peer review

Not commissioned, externally peer-reviewed.

## References

[1] Sun YQ Zhong Q Lv CB. The safety and efficacy of neoadjuvant immunochemotherapy following laparoscopic gastrectomy for gastric cancer: a multicenter real-world clinical study. Int J Surg 2024; [Epub ahead of print] 10.1097/JS9.0000000000001468 PMC11326023 38652275

[2] Nico R Veziant J Chau A. Optimal lymph node dissection for gastric cancer: a narrative review. World J Surg Oncol 2024;22:108. 38654357 10.1186/s12957-024-03388-4 PMC11036764

[3] Li K Leng X He W. Resected lymph nodes and survival of patients with esophageal squamous cell carcinoma: an observational study. Int J Surg 2023;109:2001–2009. 37222685 10.1097/JS9.0000000000000436 PMC10389544

[4] Wu H Ma W Jiang C. Heterogeneity and adjuvant therapeutic approaches in MSI-H/dMMR resectable gastric cancer: emerging trends in immunotherapy. Ann Surg Oncol 2023;30:8572–8587. 37667098 10.1245/s10434-023-14103-0 PMC10625937

[5] Kakeji Y Yoshida K Kodera Y. Three-year outcomes of a randomized phase III trial comparing adjuvant chemotherapy with S-1 plus docetaxel versus S-1 alone in stage III gastric cancer: JACCRO GC-07. Gastric Cancer 2022;25:188–196. 34351555 10.1007/s10120-021-01224-2

